# Trends in gender and ethnic diversity among US psychiatry residents: A 5‐year analysis

**DOI:** 10.1002/pcn5.70023

**Published:** 2024-10-14

**Authors:** Ahmad Abu‐Zahra, Shady Shebak, Cameron Risma

**Affiliations:** ^1^ Michigan State University College of Human Medicine East Lansing Michigan USA; ^2^ Department of Psychiatry Michigan State University College of Human Medicine East Lansing Michigan USA; ^3^ Pine Rest Christian Mental Health Services Grand Rapids Michigan USA

## Abstract

This study aims to analyze trends in gender and ethnic diversity among incoming US psychiatry residents over the past 5 years. Physician diversity improves patient care as minority physicians often serve underserved communities and address health disparities. Increasing the representation of women and underrepresented minorities in medicine is essential for preparing a physician workforce that can effectively care for an increasingly diverse population. This study highlights the importance of efforts to enhance diversity within the field of psychiatry.

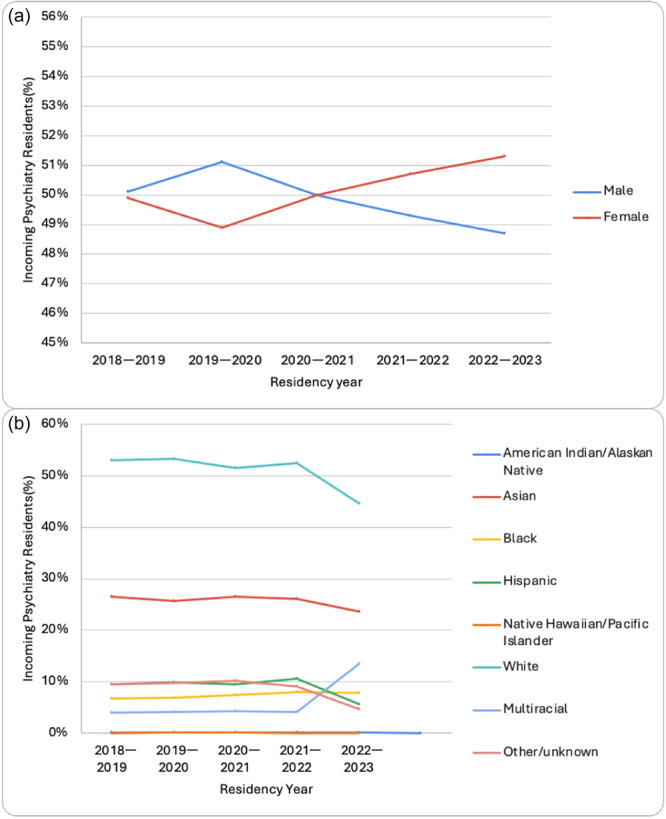

This study aims to analyze trends in gender and ethnic diversity among incoming US psychiatry residents over the past 5 years. Physician diversity improves patient care as minority physicians often serve underserved communities and address health disparities. Increasing the representation of women and underrepresented minorities in medicine is essential for preparing a physician workforce that can effectively care for an increasingly diverse population. This study highlights the importance of efforts to enhance diversity within the field of psychiatry.

As the United States approaches the demographic milestone of minorities becoming the majority by 2044, the need for a diverse physician workforce becomes paramount.^1^ Diverse physicians have been shown to improve patient care outcomes, particularly by serving underserved populations and contributing to disparities in medical research.^2^ An increased presence of underrepresented minorities (URMs) in the medical field is vital to ensuring the physician workforce can properly reflect and address the needs of the increasingly diverse population.^3^

This study will investigate the trends in gender and ethnic representation among US psychiatry residents over the past 5 years, highlighting the advancements and ongoing challenges in diversifying this critical area of healthcare. We focused on psychiatry residents due to the field's increasing competitiveness and the critical role diversity plays in addressing mental health disparities.^4^

We analyzed gender and ethnic data from the Graduate Medical Education (GME) Census,^5^ representing US psychiatry residents. We used Microsoft Excel to assess gender and URM trends from 2018–2019 to 2022–2023. Statistical tests were not used; we analyzed yearly percentage trends.

Residents self‐reported their gender as either female or male and their ethnicity as American Indian or Alaskan Native, Native Hawaiian or Pacific Islander, Hispanic or Latino, Black, Asian, White, Other or Unknown, or Multiracial.

Figure 1 displays the gender and ethnic trends among incoming psychiatry residents from 2018–2019 to 2022–2023. Incoming Black psychiatry residents showed a slight increase from 6.78% in 2018 to 7.82% in 2022. Hispanic residents showed a similar increase from 9.44% to 10.53% over the same period. American Indian or Alaskan Native residents remained low, ranging from 0.01% to 0.15%, showing a significant relative increase. Native Hawaiian or Pacific Islander residents showed minimal representation, decreasing from 0.12% to 0%. Incoming Asian residents also remained stable, decreasing slightly from 26.57% to 23.66%. Conversely, White residents decreased from 52.96% in 2018–2019 to 44.60% in 2022–2023. In 2018–2019, males held 50.10% of positions, slightly outnumbering females. By academic year 2022–2023, the proportion of female psychiatry residents rose to 51.30%, with females surpassing males in 2020–2021, reflecting diversity efforts. White residents declined, while Asian, Hispanic, and Black residents remained stable. URMs, like Native Americans and Pacific Islanders, are still underrepresented, supporting the need for further diversity efforts in psychiatry.

**Figure 1:**
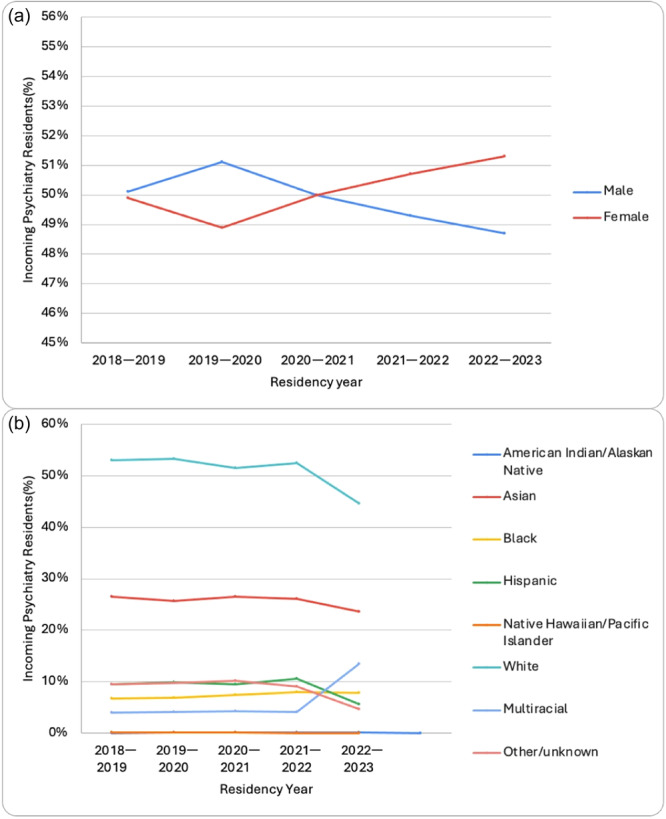
(a) Gender and (b) ethnic trends in incoming US psychiatry residents from 2018–2019 to 2022–2023.

This study assessed the trends in female and URM representation among incoming psychiatry residents from 2018 to 2022. While the overall number of incoming residents increased from 6014 to 7053, the percentages of URMs, including Black, Hispanic, American Indian or Alaskan Native, and Native Hawaiian or Pacific Islander groups, remained relatively low. Female representation increased slightly from 49.90% to 51.30% between 2018 and 2022. The rise in female psychiatry residents reflects broader trends, with increasing female representation across most specialties. Female representation has declined in other specialties such as plastic surgery, ophthalmology, and physical medicine.

Enhancing diversity in all areas of medical training, including psychiatry residencies, is imperative. In psychiatry, understanding the diverse cultural and life experiences among patients is crucial for delivering effective care and treatment. URM underrepresentation may stem from barriers to advancement and racial bias.^6^, ^7^ Additionally, the lack of role models and mentors from similar backgrounds may discourage potential URM candidates from pursuing careers in psychiatry which is a challenge that needs to be addressed to ultimately enhance patient outcomes and diversify the healthcare workforce.^8^ Limitations of this study include a small sample size, short time frame from 2018–2022, and nonparticipation by some programs in the GME census.

The underrepresentation of minorities in psychiatry impacts mental health research, patient care, and service access.^8^ It is imperative to tackle these issues to foster equity and inclusivity within psychiatry, ultimately leading to improved patient outcomes and a diverse and representative healthcare workforce.^9^ Female representation has improved, but minority groups, especially American Indian and Pacific Islander residents, remain underrepresented. Future efforts should focus on diversifying the workforce. We aspire for this study to have an impact on meaningful discussions and actions in promoting diversity in psychiatry and the healthcare workforce in general.

## AUTHOR CONTRIBUTIONS

A.A. was involved in conceptualization; data curation; formal analysis; funding acquisition; investigation; methodology; project administration; resources; software; supervision; validation; visualization; writing—original draft; writing—review and editing. C.R. was involved in conceptualization; formal analysis; project administration; validation; writing—original draft; writing—review and editing.

## CONFLICT OF INTEREST STATEMENT

The authors declare no conflict of interest.

## ETHICS APPROVAL STATEMENT

N/A.

## PATIENT CONSENT STATEMENT

N/A.

## CLINICAL TRIAL REGISTRATION

N/A.

## Data Availability

Data sharing is available for this article upon reasonable request to the corresponding author.
